# Anecdote of General Washington

**Published:** 1864-12

**Authors:** Laurie Todd


### ANECDOTE OF GENERAL WASHINGTON.

BY LAURIE TODD.

In 1796 I heard the farmer referred to narrate the following incident: "When the British army had possession of New York, and Washington with the American army was encamped near West Point, I went out one morning at sunrise to bring home the cows; on passing a clump of brushwood I heard a moaning sound, as from a person in distress. On nearing the spot I heard the words of a man at prayer. I listened from behind a tree. When he came forth I found it was Washington." This farmer was a member of the Society of Friends, who, being opposed to war under any pretext, were lukewarm, and in some cases opposed the Revolution. This man was himself a Tory. Having seen the General enter the camp, he went to his own house and said to his wife: "Martha! we must not oppose this war any longer. This morning I heard the man George Washington send up a prayer to Heaven for his country, and I know it will be heard."

This Friend dwelt between the lines of the two armies, and subsequently gave Washington many items concerning the movements of the enemy which rendered good service to the American cause. From this incident it may be inferred that Washington rose with the sun to pray for his country, fought for her at meridian, and watched for her at midnight.

